# 2D-perfusion angiography for intra-procedural endovascular treatment response assessment in chronic mesenteric ischemia: a feasibility study

**DOI:** 10.1186/s12880-022-00820-7

**Published:** 2022-05-16

**Authors:** Annette Thurner, Anne Marie Augustin, Thorsten Alexander Bley, Ralph Kickuth

**Affiliations:** Department of Diagnostic and Interventional Radiology, University Hospital Würzburg, Oberdürrbacher Str. 6, 97080 Würzburg, Germany

**Keywords:** 2D-perfusion angiography, Chronic mesenteric ischemia, Endovascular treatment, Mesenteric stenting

## Abstract

**Background:**

Endovascular revascularization has become the first-line treatment of chronic mesenteric ischemia (CMI). The qualitative visual analysis of digital subtraction angiography (DSA) is dependent on observer experience and prone to interpretation errors. We evaluate the feasibility of 2D-Perfusion Angiography (2D-PA) for objective, quantitative treatment response assessment in CMI.

**Methods:**

49 revascularizations in 39 patients with imaging based evidence of mesenteric vascular occlusive disease and clinical signs of CMI were included in this retrospective study. To assess perfusion changes by 2D-PA, DSA-series were post-processed using a dedicated, commercially available software. Regions of interest (ROI) were placed in the pre- and post-stenotic artery segment. In aorto-ostial disease, the inflow ROI was positioned at the mesenteric artery orifice. The ratios outflow to inflow ROI for peak density (PD), time to peak and area-under-the-curve (AUC) were computed and compared pre- and post-interventionally. We graded motion artifacts by means of a four-point scale. Feasibility of 2D-PA and changes of flow parameters were evaluated.

**Results:**

Motion artifacts due to a mobile vessel location beneath the diaphragm or within the mesenteric root, branch vessel superimposition and inadequate contrast enhancement at the inflow ROI during manually conducted DSA-series via selective catheters owing to steep vessel angulation, necessitated exclusion of 26 measurements from quantitative flow evaluation. The feasibility rate was 47%. In 23 technically feasible assessments, PD_outflow_/PD_inflow_ increased by 65% (p < 0.001) and AUC_outflow_/AUC_inflow_ increased by 85% (p < 0.001). The time to peak density values in the outflow ROI accelerated only minimally without reaching statistical significance. Age, BMI, target vessel (celiac trunk, SMA or IMA), stenosis location (ostial or truncal), calcification severity, plaque composition or the presence of a complex stenosis did not reach statistical significance in their distribution among the feasible and non-feasible group (p > 0.05).

**Conclusions:**

Compared to other vascular territories and indications, the feasibility of 2D-PA in mesenteric revascularization for CMI was limited. Unfavorable anatomic conditions contributed to a high rate of inconclusive 2D-PA results.

## Introduction

In chronic mesenteric ischemia (CMI), occlusive disease affecting at least two of three mesenteric arteries causes inadequate blood supply to meet the metabolic demands of the intestine [1]. The prevalence of CMI is high in atherosclerotic patients and insidious, often non-specific clinical symptoms hamper timely diagnosis. Patients suffer from ischemic gastro- or enteropathy with malabsorption, usually manifesting as angina abdominalis with postprandial pain, nausea, diarrhea, gastrointestinal bleeding and unintended weight loss [2].

Main goals of revascularization are symptom relief, improvement of the nutritional status and avoiding progression to acute-on-chronic mesenteric ischemia with bowel infarction [1, 3]. Percutaneous, minimally invasive endovascular therapy with stent placement has become the preferred first-line revascularization treatment of CMI [3–9].

During stenting of stenotic mesenteric arteries, the subjective, qualitative visual analysis of monochromatic digital subtraction angiography (DSA) images in the splanchnic territory is challenging. In general, evaluation of disease extent and treatment success in DSA depends on observer experience and is prone to perceptual bias and interpretation errors, leading to a high degree of inter-observer variability and poor reproducibility [10]. Especially in the splanchnic territory, severe aorto-ostial or non-aorto-ostial vessel calcification, vessel tortuosity and angulation may considerably impede procedure planning and visualization of treatment results. In addition, motion artifacts due to breathing or bowel movement with superimposition of bowel gas may substantially limit image interpretation. Furthermore, compared to other vascular regions, radiography of the visceral artery territory requires a larger target volume to be examined and fluoroscopic image quality may be reduced. Therefore, any assistance in procedure planning and treatment success evaluation is welcome.

In different vascular territories and indications, 2D-perfusion angiography (2D-PA) proved to be a useful tool providing on-table visualization of a complete DSA series in a color-coded contrast flow chart as well as objective, intra-procedural quantification of flow changes [11–17]. Nevertheless, 2D-PA in endovascular CMI treatment has not been addressed yet. Therefore, the aim of our study was to evaluate the feasibility of 2D-PA for endovascular treatment assessment in CMI.

## Material and methods

### Study design and patient characteristics

In total, 49 mesenteric endovascular revascularizations in 39 consecutive patients suffering from CMI were included in this retrospective, single-center observational case–control study. Our study group consisted of 21 men and 18 women with a median age of 69.7 (range 48–87 years), scheduled for endovascular revascularization at our tertiary care hospital from August 2013 to August 2021. At presentation, the leading clinical symptom consisted of recurrent abdominal pain immediately after a meal or, in one case, triggered by hemodialysis-associated hypotension. As a result, the majority of our study population complained unintended weight loss due to food fear and malabsorption. The patients’ weight loss ranged between 7 kg in 48 weeks to 10 kg in 4 weeks. The median BMI was 19.3 kg/m^2^ (range 14.9–34.5). Additionally, 38% of our patients presented with endoscopically proven ischemic gastritis or colitis with intestinal bleeding episodes. The other patients had normal or inconclusive endoscopic results with regard to gastric or colonic ischemic mucosal injury. We present the patients’ demographics and clinical symptoms in Table 1.

**Table 1:** Patients’ demographic and clinical characteristics

Number of patients	39
Number of procedures	44
Number of treated arteries	49
**Patients’ demographics**	
Median age (years ± standard deviation)	69.7 ± 9.8
Gender (n, %)	
Male	21 (54%)
Female	18 (46%)
BMI (kg/m^2^ ± standard deviation)	19.3 ± 10.3
History of (n, %)	
Hypertension	38 (97%)
Heavy smoking [> 20 pack years]	27 (69%)
Diabetes	21 (53%)
Coronary artery disease ± coronary bypass surgery	27 (69%)
Stroke	2 (5%)
Peripheral artery disease	28 (72%)
Chronic kidney disease stage 1 (GFR > 90 mL/min/1.73m^2^)	7 (18%)
Chronic kidney disease stage 2 (GFR 60–89 mL/min/1.73m^2^)	13 (33%)
Chronic kidney disease stage 3 (GFR 30–59 mL/min/1.73m^2^)	12 (31%)
Chronic kidney disease stage 4 + 5 (GFR < 29 mL/min/1.73m^2^ or dialysis)	7 (18%)
Anemia (hemoglobin < 13.5 g/dl; hematocrit < 40%)	30 (77%)
**Clinical characteristics**	
Clinical symptoms at presentation (n, %)	
Abdominal pain (postprandial)	39 (100%)
Weight loss	36 (92%)
Ischemic gastritis/colitis ± bleeding	15 (38%)
Immediate post-interventional pain decrease per procedure (n, %)	42 (95%)
Number of patients lost to follow-up after discharge (n, %)	11 (28%)
Weight gain per patient in follow-up (n, %)	21 (75%)
Recurrence of symptoms per patient in follow-up (n, %)	12 (43%)
Number of recorded deaths in follow-up (n, %)	7 (25%)
Bowel-infarction-related death in follow-up (n, %)	4 (14%)

*BMI* body mass index, *GFR* glomerular filtration rate

Moreover, prerequisite for inclusion was imaging based evidence of occlusive visceral artery disease of atherosclerotic origin in abdominal computed tomographic (CT) or magnetic resonance (MR) imaging. In symptomatic patients, we considered a more than 70% stenosis in single-vessel disease or a more than 50% stenosis in multi-vessel disease relevant [18]. Preprocedural evaluation of the target visceral arteries with computed tomography revealed moderate (25–50% circumference) to severe (> 50% circumference) vessel wall calcification in more than 85% [19]. In computed tomographic imaging, 33% of treated arteries showed calcified plaques, 10% had soft plaques and 57% had a mixed plaque composition. We found triple artery disease, affecting celiac trunk, superior mesenteric artery (SMA) and inferior mesenteric artery (IMA) in 22 patients (including one patient with celiacomesenteric trunk) and double vessel disease in 16 patients. Only one patient had a single vessel disease with a truncal SMA stenosis of more than 70%. In this case, the patient suffered from recurrent severe abdominal pain during hemodialysis, triggered by hemodialysis-associated hypotension. He was on dialysis for five years and had multiple cardiovascular comorbidities (e.g., diabetes mellitus type 2, coronary artery disease with ischemic cardiomyopathy and history of coronary-bypass surgery). Thus, evidence of a diffuse atherosclerotic disease with insufficient collateral flow via the mesenteric arcades induced us to intervene in this particular case. 88% of target arteries had aorto-ostial stenosis. In 22% of our interventions, we found complex visceral artery stenosis with a stenosis length of more than 20 mm and heavily calcified lesions including complete occlusions (maximum stenosis length 40 mm). Compared to DSA, noninvasive imaging had high accuracy in revealing stenoses of > 50% or > 70%, respectively [20]. We provide an overview of the patients’ visceral artery disease distribution in Table 2.

**Table 2:** Visceral artery disease, procedure and 2D-PA characteristics

Number of patients	39
Number of procedures	44
Number of treated arteries	49
**Visceral artery disease characteristics**	
Visceral artery disease distribution per patient (n, %)	
Triple vessel	22 (56%), incl. 1 celiac-mesenteric trunk
Double vessel	16 (41%)
Single vessel	1 (3%)
Stenosis distribution per treated vessel (n, %)	
Ostial	43 (88%)
Truncal	6 (12%)
Complex stenosis (stenosis length > 20 mm long, and/or heavily calcified or severely irregular lesions, occlusions)	11 (22%)
Stenosis length (mm)	5–40 (mm)
Target vessel calcification severity per treated vessel (n, %)	
Mild (< 25% circumference)	7 (14%)
Moderate (25–50%)	12 (25%)
Severe (> 50% circumference)	30 (61%)
Computed tomographic plaque composition per treated vessel (n, %)	
Soft	5 (10%)
Mixed	28 (57%)
Calcified	16 (33%)
**Procedure and 2D-PA characteristics**	
Revascularization per procedure (n, %)	
Single vessel	40 (91%)
Superior mesenteric artery (SMA)	26 (65%), incl. 1 celiac-mesenteric trunk
Celiac Trunk	11 (28%)
Inferior mesenteric artery (IMA)	3 (7%)
Double vessel (SMA + Celiac Trunk)	3 (7%)
Triple vessel (SMA + Celiac Trunk + IMA)	1 (2%)
Stent diameter (mm) / Stent length (mm)	4–8 (mm) / 12–27 (mm)
Bare-metal stenting per treated vessel	39 (80%)
Covered-stenting per treated vessel	5 (10%)
PTA alone per treated vessel	5 (10%)
Technical failure (n, %)	0 (0%)
Major complications per procedure (n, %)	3 (7%)
2D-PA measurements performed (n, %)	49 (100%)
2D-PA motion artifact scale per treated artery (n, %)	
1: None	0 (0%)
2: Mild	17 (35%)
3: Moderate	24 (49%)
4: Severe	8 (16%)
Conclusive 2D-PA hemodynamic result (n, %)	23 (47%)

*2D-PA* 2D-perfusion angiography,* PTA* percutaneous transluminal angioplasty

Our interdisciplinary conference of vascular surgeons and interventional radiologists approved the indication for endovascular visceral revascularization in each case. In addition, we reviewed clinical and imaging records of each case in our Clinical Information System and Picture Archive and Communication System to collect further information about the patients’ medical history and the post-procedure follow-up of median 21 months (range: 4 months to 7 years). 

### Procedure technique

One interventional radiologist with 25 years post-certification experience performed the visceral revascularizations via a trans-femoral access route, using a monoplane, ceiling-mounted angiographic system (Axiom Artis Zee, Siemens, Forchheim, Germany). In two cases, catheter instability due to iliac artery elongation and an acute angle between target vessel and aorta necessitated switching to a trans-brachial approach. At least, one stenotic or occluded mesenteric artery had to be recanalized.

The mesenteric target vessels were examined by selective manual angiograms in laterally angled, parasagittal views using different 5 F catheter configurations and iodinated contrast media (Imeron 300; Bracco Imaging, Konstanz, Germany). In order to visualize the extent of aorto-ostial stenosis, a 0.018-inch guidewire was placed through the selective catheter distally to the stenotic segment and a angiogram was performed with the catheter tip being retracted into the aortic lumen next to the mesenteric artery orifice. Intra-arterial contrast was administered manually by a single operator before and after mesenteric revascularization via selective catheters or long guiding sheaths. For separate pre- and post-interventional hand injection, we used a flow rate of approximately 8 ml/s and a 1:1 normal saline dilution of contrast media (Imeron 300) for each patient regardless of patient size. Only in a few cases, pump-assisted selective angiograms were conducted with 20–30 ml of contrast (80–100% Imeron 300 dilution) and a flow rate of 6–7 ml/s (3 s inject delay, 0 s X-ray delay, 600 psi, injection time 2.9–5 s; Accutron HP-D, Medtron AG, Saarbrücken, Germany). The parameters were chosen at the operator’s discretion according to the target vessel size and the patients’ renal function.

Due to impaired renal function and previously performed diagnostic CT- or MR-angiographic images, we waived pump-assisted aortography in the majority of cases. DSA images were acquired at 2 frames per second with a range of approximately 5–6 s per DSA run.

During the procedure, we did not administer spasmolytic agents.

We assessed major complications according to the reporting standards of the Society of Interventional Radiology [21]. Table 2 includes details of the mesenteric stenting procedure.

### Postprocessing and 2D-PA analysis

To evaluate perfusion changes by 2D-PA, we performed post-processing of monochromatic DSA series before and after mesenteric stenting on a dedicated workstation using a commercially available clinical software application (Syngo X Workplace VB20D, Syngo iFlow software, Siemens, Forchheim, Germany). In 2D-PA, a single computed, color-coded composite image provides clustered information about vessel morphology and contrast concentration changes per time during a complete DSA run. In addition, 2D-PA allows calculation of flow curves and blood flow parameters in user-defined regions of interest (ROI). Therefore, 2D-PA enables an objective assessment of flow changes and perfusion parameters.

Initially, we selected pre- and post-interventional DSA series taken in almost identically angled views to be evaluated by 2D-PA. The chosen DSA series were prepared for 2D-PA-analysis by automatic isocenter calibration (calibration factor 0.2017 (mm/Pixel)) according to the instructions for use. Next, in total four ROI were placed, at least one proximally and one distally to the stenotic mesenteric artery segment. ROIs were arranged in the same position pre- and post-interventionally, to ensure measurements at comparable locations. ROIs were adjusted to at least 2/3^rd^ of the vessel diameter. In cases of aorto-ostial disease, we placed the inflow ROI in the aortic lumen next to the mesenteric artery ostium. We located the outflow ROI, intended for statistical analysis, at least 2 cm distally to the stenotic segment. The reported 2D-PA technique using a two ROI based evaluation (a proximal reference ROI and a distal target ROI) is independent of a standardized contrast-bolus protocol [16].

In 2D-PA, the computed time-versus-ROI-contrast-intensity result tables offer the following numeric parameters: ROI Area in mm^2^, the ratio between outflow and inflow maximal contrast density per ROI after contrast media application (ROI Peak/Ref Peak or PD_outflow_/PD_inflow_). Furthermore, the time to peak density per ROI in seconds from the first moment of increase of density to the maximum contrast density (ROI Peak time or TTP) and the ratio between outflow and inflow area-under-the-time-density-curve (ROI AUC/Ref AUC or AUC_outflow_/AUC_inflow_).

In addition, we calculated the ratio between outflow and inflow time to peak in order to overcome variations in the flow rates between consecutive manual angiograms.

Additionally, we assessed the 2D-PA-images qualitatively for motion artifacts during a DSA run. To this end, we graded motion artifacts by means of a four-point scale (Score 1: artifact-free; Score 2: minimal motion-related artifact with no effect on flow evaluation; Score 3: moderate motion-related artifact with some, but not severe effect on diagnostic quality; Score 4: severe motion-related artifact rendering flow evaluation impossible). Furthermore, we evaluated the time versus contrast density curves with regard to conclusiveness of hemodynamics. We considered results hemodynamically conclusive, if an adequate contrast enhancement is present at the inflow ROI during the DSA run.

In theory, following successful revascularization, the TTP-ratio (TTP_outflow_/TTP_inflow_) decreases, since the density peak in the outflow ROI will be reached earlier. The peak density-ratio (PD_outflow_/PD_inflow_) increases, since the maximal contrast density in the outflow ROI rises following intervention. Correspondingly, the AUC-ratio (AUC_outflow_/AUC_inflow_) increases as well, since the outflow AUC approximates the inflow AUC post-interventionally.

### Endpoint definition

We designated technical feasibility as primary endpoint. Technical feasibility was defined as successful completion of the post-processing process, freedom from severe motion artifacts or vessel superimposition and conclusiveness of hemodynamic results. Secondary endpoint for 2D-PA was defined as statistically significant changes of flow parameters pre- and post-interventionally indicating treatment success.

### Statistical analysis

Microsoft Excel (Microsoft Office 365 ProPlus, Version 1803) served as a data collection tool. For statistical analysis, we used a dedicated software (R®, Version 4.0.2, The R Foundation for Statistical Computing, Wien, Austria). Descriptive data were presented as means ± standard deviation for normally distributed variables or medians with ranges for non-normally distributed variables. We reported categorical variables as frequencies and percentages with n (%). The Anderson Darling test was applied for assessment of normality, rejecting the hypothesis of normality when the p-value was less or equal to 0.05. Ratios of peak density, time-to-peak and area under the time-contrast curve pre- and post-interventionally were evaluated for statistical significance by using the pairwise Wilcoxon´s signed rank test for non-normally distributed variables. In addition, we assessed whether parameters, potentially influencing the feasibility, were more frequently represented in the feasible- or non-feasible group by using the fisher’s exact test for nominally scaled variables, the Wilcoxon´s signed rank test for ordinally scaled variables and the T-test for scaled, normally distributed variables. A p-value of less than 0.05 was designated as statistically significant.

## Results

**Fig. 1:**
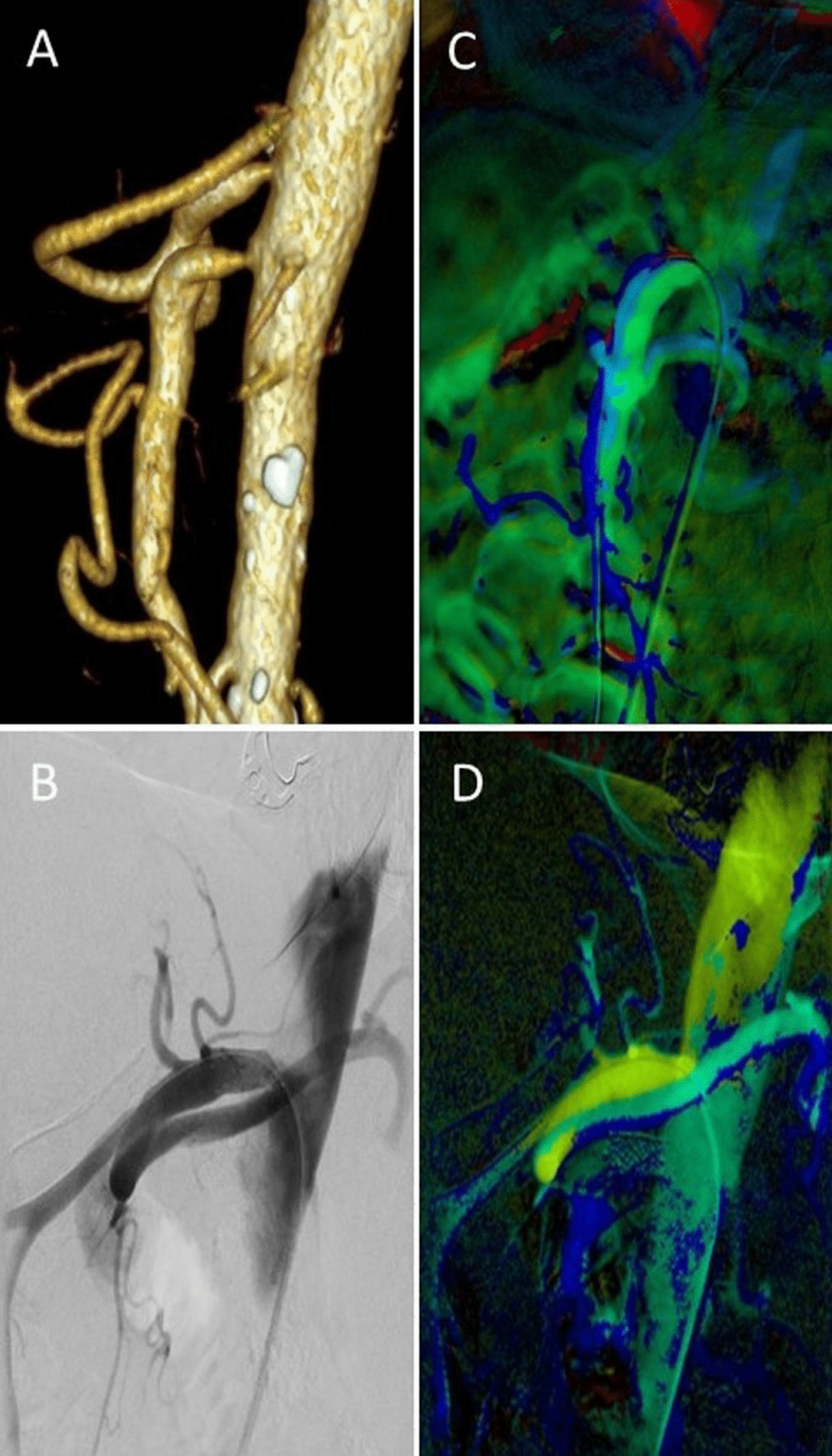
Impaired 2D perfusion assessment in a patient with 2-vessel-disease. Preinterventional Volume Rendering CT Angiography image (**A**), digital subtraction angiogram and 2D perfusion images showing vessel superimposition due to angulation of the celiac trunk (**B**, **D**) and blurring of vessel contours due to breathing artifacts in SMA and celiac trunk (**C**, **D**)

**Fig. 2:**
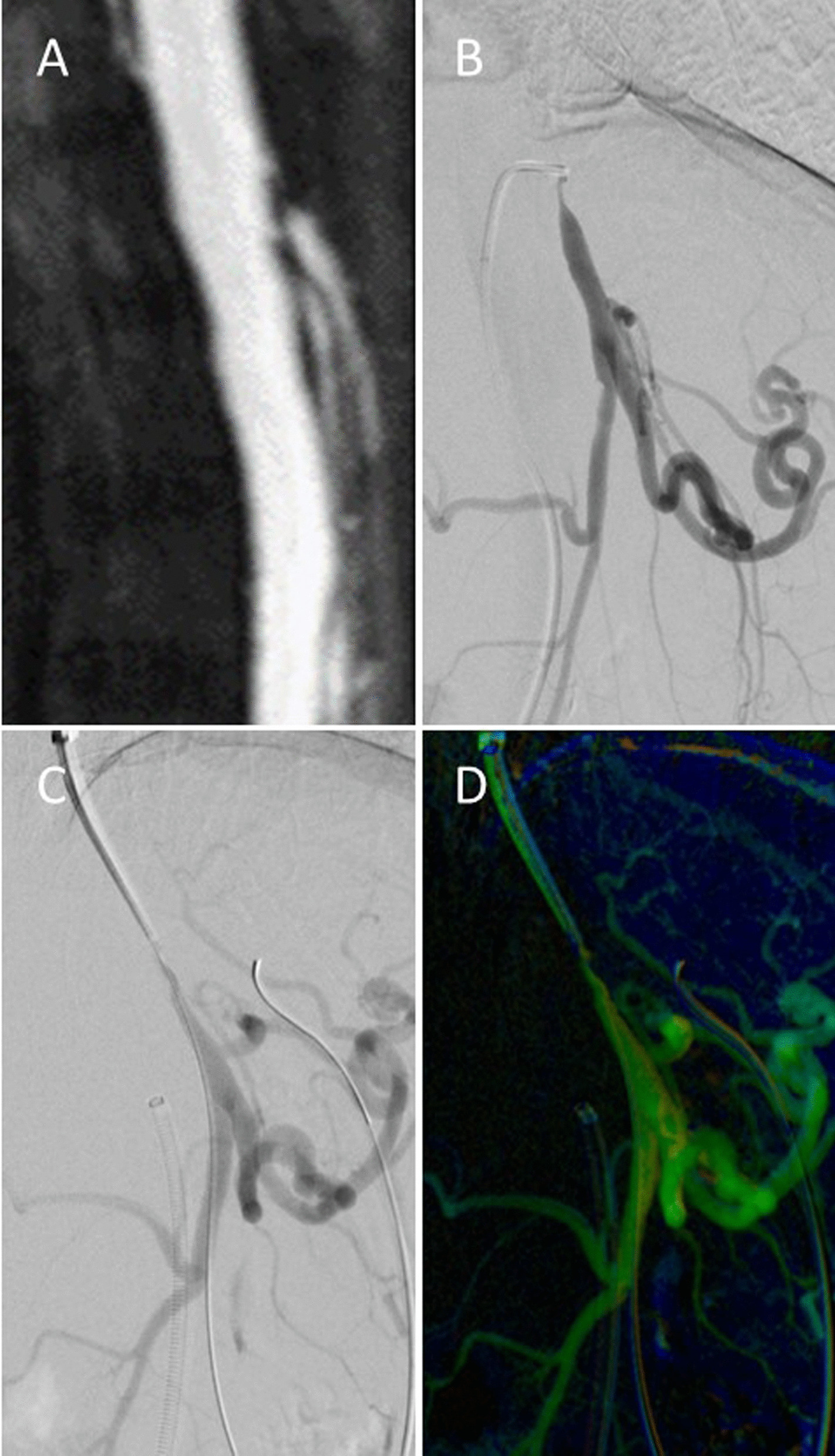
Preinterventional maximum intensity projection MR-angiography image (**A**), transfemoral digital subtraction angiogram (**B**), transbrachial digital subtraction angiogram and 2D perfusion image (**C**, **D**) in a patient with filiform ostial stenoses of celiac trunk and SMA. Acute angulation of the celiac trunk to the aorta coupled with transfemoral instability of the catheter system necessitated switching to a transbrachial access route. Insufficient prestenotic aortic contrast enhancement impeded 2D perfusion evaluation via selective catheter angiogram. Due to renal insufficiency, we waived aortography in this case

Completion of the post-processing process was achieved in each case. With regard to motion artifacts, we recorded mild to moderate artifact levels in 84% of evaluated arteries. In 16% severe motion artifacts rendered adequate flow evaluation impossible. Artifacts were due to a combination of the patients’ inability to hold their breath during a DSA run and bowel movement. In five cases, vessel branching and tortuosity precludes free projection of the intended inflow or outflow vessel segments for ROI placement (Fig. 1). Indeed, in each non-feasible case, insufficient prestenotic contrast enhancement during a manually conducted DSA series via long guiding sheaths or selective catheters was the main reason for impeded 2D-PA assessment (Fig. 2).

As a result, hindered 2D-PA due to motion artifacts, vessel superimposition and inadequate pre-stenotic contrast enhancement necessitated exclusion of overall 26 2D-PA measurements from quantitative flow evaluation. Therefore, the technical feasibility rate of 2D-PA in mesenteric stenting was 47%. Further 2D-PA results are included in Table 2.

Nevertheless, we recorded the following changes of flow parameters pre- and post-interventionally in 23 technically feasible, conclusive 2D-PA measurements: PD and AUC in the target outflow ROI were significantly higher following treatment, resulting in an increase of 65% for PD_outflow_/PD_inflow_ (p < 0.001) and 85% for AUC_outflow_/AUC_inflow_ (p < 0.001). The time to peak density values in the outflow ROI following mesenteric stenting accelerated only minimally without reaching statistical significance (p = 0.553). The decrease in the TTP_outflow_/TTP_inflow_ ratio pre- and post-interventionally was -2%. The correlation between the increase of AUC_outflow_/AUC_inflow_ and the post-procedural weight gain yielded statistical significance (p = 0.016).

**Fig. 3:**
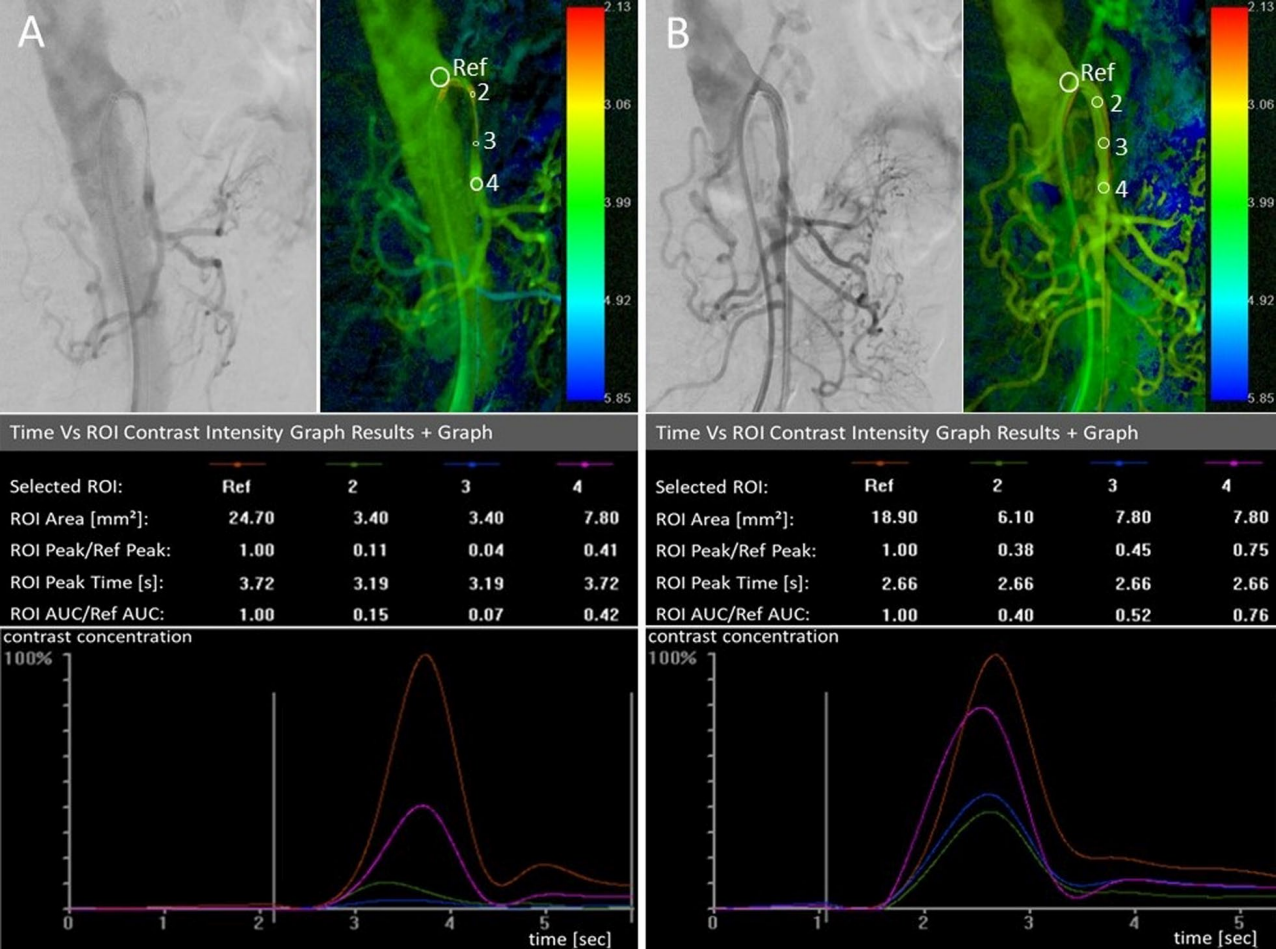
2D perfusion angiography evaluation in a patient with truncal SMA stenosis and ostial celiac trunk occlusion. Digital subtraction angiogram, color-coded 2D perfusion image and time versus contrast-concentration graph pre-interventionally (**A**) and following endovascular therapy with stenting (**B**). Post-interventionally, vessel diameters in the visceral territory increased substantially and the retrograde collateral flow to the celiac territory markedly improved. Accordingly, color-coded hemodynamics changed to a slightly warmer tone and flow parameters inferred a higher contrast concentration distal to the treated stenosis from an increase of AUC- and PD-ratio. On the contrary, the measurements failed to show the expected decrease in TTP-ratio

Moreover, a subgroup analysis of variables’ impact on the feasibility of 2D-PA yielded no statistical significance in the majority of cases. Age (p = 0.475), BMI (p = 0.343), target vessel (celiac trunk, SMA or IMA; p = 0.234), stenosis location (ostial/truncal; p = 0.086), calcification severity (p = 0.187), plaque composition (p = 0.639) or the presence of a complex stenosis (p = 0.086) did not reach statistical significance in their distribution among the feasible and non-feasible group. A higher artifact level (p = 0.006) and a hemodynamically inconclusive result (p < 0.001) were found more often in the non-feasible group by reaching high statistical significance. Changes in 2D-PA following mesenteric stenting are presented in Table 3, and Fig. 3 offers an example of 2D-PA before and after mesenteric stenting.

**Table 3:** Statistical 2D perfusion angiography evaluation (n = 23 conclusive measurements)

	Pre-intervention median value (range)	Post-intervention median value (range)	Delta pre- and post-intervention (%)	p value
PD_outflow_/PD_inflow_	0.60 (0.17–2.01)	0.99 (0.28–3.88)	+ 0.39 (+ 65%)	< 0.001
TTP_outflow_/TTP_inflow_	1.01 (0.53–1.39)	0.99 (0.79–1.17)	− 0.02 (− 2%)	0.553
AUC_outflow_/AUC_inflow_	0.59 (0.19–1.79)	1.09 (0.28–3.55)	+ 0.5 (+ 85%)	< 0.001

*PD* peak density, *TTP* time to peak, *AUC* area-under-the-time-density-curve

## Discussion

This study adds further aspects to the growing experience in 2D-PA by showing that 2D-PA in the mesenteric territory for treatment evaluation in chronic mesenteric ischemia is challenging. Non-interpretable 2D-PA evaluations necessitated exclusion of 26 measurements from quantitative flow evaluation, resulting in a feasibility rate of 47%. Previously published studies indicated 2D-PA as highly susceptible to motion artifacts, since they cause inaccurate measurements of perfusion parameters. Indeed, in comparison with previously published studies, our feasibility rate was considerably lower. For instance, 2D-PA in peripheral artery disease proved to be technically feasible in all cases reported by Hinrichs et al. and Dewald et al. [16, 17]. Furthermore, Maschke et al. reported no patient exclusion due to hindered 2D-PA assessment in TIPS-revision and an exclusion rate of 13% in 2D-PA assessment following transarterial liver chemoembolization [13, 14]. In a small patient collective with non-occlusive mesenteric ischemia (NOMI), only 6% of patients had to be excluded due to inadequate 2D-PA analysis [15].

Our results may be triggered by additional challenges in occlusive mesenteric artery disease. First, the mobile vessel location beneath the diaphragm or within the mesenteric root, rendered 2D-PA vulnerable to motion artifacts. Since we did not apply spasmolytic agents during our interventions, their effect on artifact reduction remains unclear. Second, especially in the celiac territory, superimposition of vessel branches precluded free projection of the intended inflow or outflow vessel segments for ROI placement.

However, the main drawback was steep angulation between the aorta and the target vessel. As a result, we recorded insufficient contrast enhancement at the inflow ROI in manually conducted angiograms via selective catheters or long guiding sheaths (Fig. 2). In these cases, 2D-PA flow curves and perfusion measurements were inconclusive. Adequate contrast enhancement at the inflow ROI is one major prerequisite for a reliable application of the reported 2D-PA technique which is based on a two ROI approach (a proximal reference ROI and a distal target ROI).

Since 49% of our patients had impaired renal function (CKD stages 3 to 5), and previously performed CT- or MR-angiographic images were available in each case, additional aortography solely for 2D-PA reasons was not justified in the majority of our cases. The fact that 2D-PA is independent of additional contrast media application and radiation exposure is one major strength of the reported 2D-PA technique.

Another key feature of the reported 2D-PA technique is “on-table” visualization of color-coded and quantitative flow changes during the procedure. To date, other promising novel imaging techniques enabling flow evaluation and/or color-coding of flow velocities such as 4D flow MRI are time consuming and not available intra-procedurally. Further, contrary to 2D-PA, they are prone to artifacts caused by dense calcifications and stent material [22]. In addition, patients with CMI are often diagnosed relatively late in the course of the disease and present with clinically decompensated CMI. Hence, the fragile status of these often multi-morbid and elderly patients may hamper the success of elaborate imaging techniques which require additional time and a high level of patient compliance.

In a subgroup analysis, none of the presumed factors influenced the feasibility significantly. Neither a higher patient age, nor a higher BMI were more frequently found in the non-feasible group. Moreover, ostial stenosis as well as the presence of complex stenosis or a higher calcification severity were not significantly differently distributed among the feasible and the non-feasible group. As expected, only a high motion artifact level and an insufficient inflow contrast enhancement proved to be highly significant limitations for 2D-PA.

In accordance with previously published data, 2D-PA evaluation in our feasible group showed a significant increase in the AUC-ratio and the PD-ratio indicating improved perfusion of the treated artery following stenting. In contrast, the TTP-ratio decreased only minimally without reaching statistical significance.

This result is in accordance with Dewald et al., who reported only a low trend towards decrease of the TTP-ratio in their assessment of 2D-PA in CO_2_-angiography of the limb [17]. The authors attributed the lack of significance to the small sample size in their study (n = 10). Additionally, in our collective, the short distance between the inflow and outflow ROI and the consecutively very short time differences may not be detectable with the 2D-PA technique in a standard DSA run at 2 frames/s. In 2D-PA, density values were assigned to each area within a ROI per DSA run. These density-over-time–values are used to deduce information about flow rates, flow time and maximum values. Hence, in a short distance the flow acceleration may insufficiently be detectable by changes in time to peak density per ROI. In view of the post-stenotic measuring point, Augustin et al. found similar results in their 2D-PA study following stenting for renal artery stenosis. In their assessment, significant TTP changes were found more distally at the level of the first-order segmental arteries, not in the main renal artery [23]. With the kidneys lying in the retroperitoneal space, 2D-PA measurements in smaller sized, segmental arteries may be less affected by motion artifacts compared to branch vessels within the mesentery. Thus, in our collective, we waived measurements at more distally located vessel branching levels within the mesenteric root, since they were prone to severe motion artifacts. Due to the retrospective design of our study, we were not able to evaluate flow changes more peripherally, e.g. in the portal vein. However, in a study by Becker et al. assessing 2D-PA in NOMI, TTP changes in the portal vein following intervention did not reach statistical significance as well [15].

Furthermore, a combination of the complex vascular anatomy with varying disease distribution patterns, the high capacity of collateral blood circulation, post-prandial flow changes, vessel wall elasticity, the intestinal vascular suction effect as well as post-occlusive reactive vasodilatation complicates the analysis of flow parameters in the mesenteric territory [24]. Previously published Doppler flow evaluations point to the complex compensatory flow changes in this territory. For instance, the IMA potentially serves as a relevant collateral vessel by offering a tenfold flow reserve in chronic SMA/celiac trunk stenosis [25]. In addition, recently published studies point to the fact that visceral flow disturbances impeding reliable flow measurements are an issue in other novel perfusion imaging techniques such as 4D flow MRI as well [26]. Even in healthy visceral arteries the curved and branched anatomy causes disturbed, vortex and/or helical flow, which hampers flow measurements [26]. Especially in combination with ostial visceral artery stenosis, oscillatory and fluctuating time velocity curves have been measured rendering flow evaluation challenging [26].

Contrary to the creatinine levels in renal artery stenting, we did not consider any clinical or laboratory parameter appropriate for statistical correlation with changes in perfusion parameters to predict the procedures’ outcome in our retrospective data set. Only the correlation between the increase of AUC_outflow_/AUC_inflow_ and the post-procedural weight gain yielded statistical significance (p = 0.016). However, the clinical relevance of this result is questionable, since the patients’ body weight depends on many factors. Six patients in our feasible group presented with symptom recurrence. Due to the small sample size and a multitude of influencing factors, correlation between changes in flow parameters and symptom relapse seems not appropriate. This study has several limitations. Due to the retrospective nature of our study, we solely focused on the feasibility of 2D-PA for intra-procedural treatment response assessment during mesenteric stenting and did not perform other techniques such as duplex sonography or pressure wire measurements to compare our 2D-PA results. As previously mentioned, there are many factors influencing the perfusion between the proximal mesenteric artery stenosis and the bowl’s capillary system, which are not gathered by the reported 2D-PA technique. Hence, 2D-PA is not able to point to absolute measurements of mesenteric perfusion. Further studies should focus on the influence of the collateral flow and techniques to evaluate the real 3D perfusion of the capillary phase of the mesenteric territories.

The small number of patients, the high number of non-interpretable 2D-PA evaluations and the moderate loss of patients available for follow up in our multi-morbid patient collective rendered a structured follow-up analysis and reliable correlation of 2D-flow parameters with clinical outcome parameters in order to define criteria for effective therapy inadequate.

## Conclusions

Compared to other vascular territories and indications, the feasibility of 2D-PA in endovascular mesenteric revascularization for CMI was limited. Unfavorable anatomic conditions contributed to a high rate of inconclusive 2D-PA results. With our data, we intended to contribute to the growing preliminary knowledge about objective treatment assessment with 2D-PA. Especially in the mesenteric territory, further prospective standardized evaluations comparing different flow measurement techniques with larger study populations and structured longitudinal follow up periods are needed to gain deeper insights in the complex mesenteric flow characteristics and to define objective standardized endpoints of successful mesenteric revascularization.

## Data Availability

The data used and analyzed are available on reasonable request. The corresponding author should be contacted if someone wants to request the data.

## Abbreviations

2D-PA: 2D-perfusion angiography
AUC: Area-under-the-time-density-curve
BMI: Body mass index
CKD: Chronic kidney disease
CMI: Chronic mesenteric ischemia
CT: Computed tomography
DSA: Digital subtraction angiography
IMA: Inferior mesenteric artery
IU: International unit
MRI: Magnetic resonance imaging
NOMI: Non occlusive mesenteric ischemia
PAD: Peripheral artery disease
PD: Peak density
PTA: Percutaneous transluminal angioplasty
ROI: Region of interest
SMA: Superior mesenteric artery
TTP: Time to peak
